# Extranodal NK/T Cell Lymphoma Causing Cardiorespiratory Failure

**DOI:** 10.1155/2016/2394809

**Published:** 2016-07-17

**Authors:** Yiting Li, Ivan Damjanov

**Affiliations:** Department of Pathology and Laboratory Medicine, University of Kansas Medical Center, 3901 Rainbow Boulevard MS 3045, Kansas City, KS 66160, USA

## Abstract

Extranodal NK/T cell lymphoma is an uncommon malignancy usually involving the sinonasal area. We report an unusual case of extranodal NK/T cell lymphoma diagnosed in a 62-year-old Caucasian male who died of progressive cardiorespiratory failure but had no clinically detectable upper respiratory system lesions. The initial diagnosis was made cytologically on a sample of pericardial fluid that contained neoplastic lymphoid cells. These cells were positive for CD2, cytoplasmic CD3, and Epstein-Barr virus and negative for CD56. The diagnosis was confirmed at the autopsy, which disclosed lymphoma infiltrates in the myocardium, lungs, stomach, and pancreas. The death was caused by heart and lung failure due to uncontrollable arrhythmia and respiratory insufficiency due to the lymphoma infiltrates. To the best of our knowledge, this is the first case of extranodal NK/T cell lymphoma presenting with cardiopulmonary failure.

## 1. Introduction

Extranodal NK/T cell lymphoma (ENKTL) is a rare type of non-Hodgkin's lymphoma that is more common in Asian and Central American countries than in North America or Europe [1]. Most of these lymphomas involve the sinonasal area only and present with a nasal mass or destruction. Only 25% of ENKTLs occur in the extranasal organs. The most commonly reported extranasal sites are the digestive tract, skin, testis, and central nervous system [2]. Cardiac, pulmonary, or pancreatic involvement is extremely rarely found and rarely if ever represents the dominant clinical manifestation. Here we report a Caucasian male American presenting with rapidly progressive cardiopulmonary failure due to ENKTL involving heart, lung, stomach, and pancreas and no definitive clinical evidence of sinonasal involvement. Lymphoma infiltrating these organs was confirmed by autopsy.

## 2. Case Presentation

**Figure 1:**
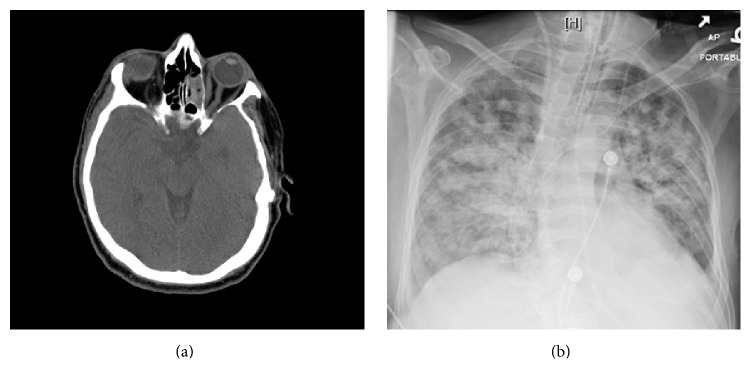
Radiologic findings. (a) Head CT scan shows fluid opacification consistent with inflammatory changes predominately in the left ethmoid sinus. No masses or bone destruction is seen. (b) Chest X-ray shows diffuse bilateral mixed interstitial and alveolar infiltrates.

A 62-year-old otherwise healthy Caucasian male complained of some minor nonspecific respiratory symptoms for a few days. These complaints evolved into a more worrisome pulmonary disease characterized by cough and progressive respiratory distress 2 days prior to the hospitalization. Chest CT scan on admission to an outside hospital showed diffuse bilateral alveolar and interstitial lung infiltrates. A diagnosis of atypical pneumonia and possible myocarditis was made and the patient was given antibiotic treatment and was discharged two weeks later. However, dyspnea persisted and he was admitted to our hospital for further workup. On admission, patient was found to have fever, acute hypoxic respiratory failure, pleural effusion, pericardial effusion, and irregular heartbeats with bouts of ventricular fibrillation. Upon hospitalization, additional diagnoses were made including acute renal failure, as well as possible liver disease marked by an elevation of liver function tests. A poorly defined pancreatic mass was noted on the CT scan. Head CT without contrast was performed which showed minimal mucosal thickening in the left maxillary sinus. There was opacification of several left ethmoid air cells with fluid levels in the left sphenoid and left maxillary sinuses. A nasoenteric tube was in place in nasal cavity. There was no mass lesion, erosion, or bone destruction. These finding were most consistent with mild inflammatory changes (Figure 1(a)).

Sepsis was suspected and he was given antibiotics without improvement of his clinical condition. His condition rapidly deteriorated with progressive dyspnea and pulmonary infiltrates which by X-ray examination involved all five lobes (Figure 1(b)). Extensive microbiological studies were performed with no positive bacteriologic, viral, or fungal isolates. The only positive test was a mildly increased DNA load of Epstein-Barr virus (EBV) in blood with 0.5 copies per microliter detected by a quantitative PCR assay. EBV capsid IgG and nuclear antigen/antibody were positive. Peripheral blood smear showed mild normocytic anemia with moderate thrombocytopenia. White cells count was within the normal range with absolute lymphopenia and no atypical lymphocytes or lymphoma cells.

As his condition deteriorated, he developed signs of cardiac tamponade. Pericardial effusion was drained and sent to the pathology laboratory. He developed uncontrollable ventricular fibrillation with subsequent cardiac arrest, which could not be reversed by resuscitation. At the same time, patient hemoglobin and platelet count decreased to 6.9 mg/dL and 22,000/*μ*L. His fibrinogen was decreased (178 mg/dL), ferritin was markedly elevated (1763 ng/Ml), and lactate dehydrogenase was elevated (628 U/L). Neutrophil count and triglyceride were within normal range. Hemophagocytic lymphohistiocytosis (HLH) was suspected. However, the final diagnosis of HLH was never made since the patient's condition deteriorated quickly and he died in a cardiopulmonary failure.

**Figure 2:**
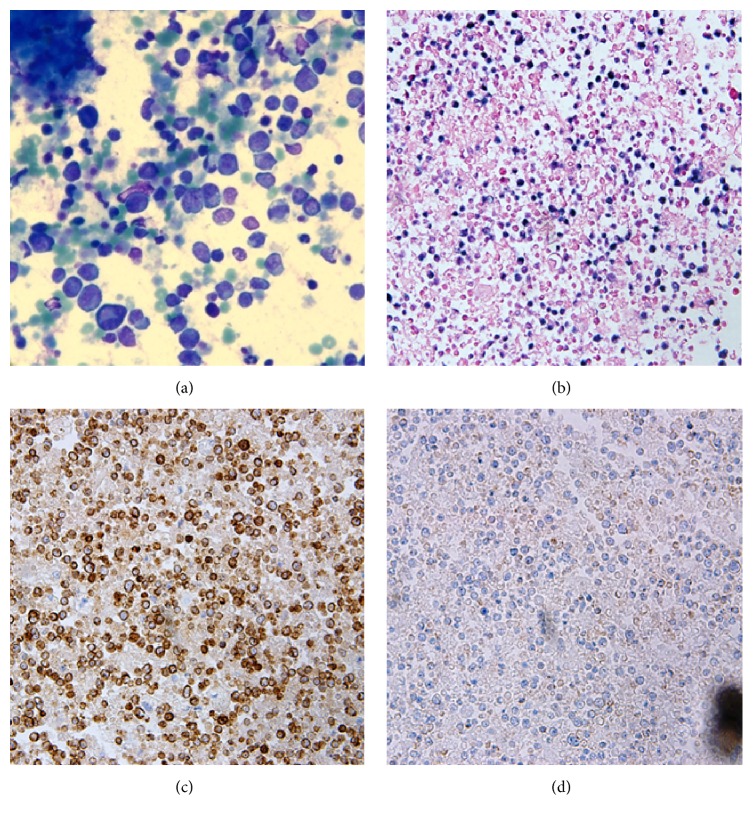
Cytology of pericardial fluid. (a) Diff-Quick stain (×400) shows numerous atypical lymphoid cells with abundant cytoplasm and irregular nuclear contour. (b) The atypical cells are strongly positive for EBV with in situ hybridization (×200). (c) Cells are also immunohistochemically positive for CD3 (×200). (d) Cells are immunohistochemically negative for CD56 (×200).

The pericardial fluid sent for cytological analysis one day before his death contained numerous markedly atypical lymphoid cells (Figure 2(a)) which were positive for Epstein-Barr virus (EBV) as detected by in situ hybridization (Figure 2(b)). Immunohistochemical stains performed on the cell block showed that the atypical cells were positive for CD45, CD2, and CD3 (Figure 2(c)) and negative for CD4, CD8, CD56 (Figure 2(d)), CD138, pan-cytokeratin, MOC-31, Ber-EP4, chromogranin, synaptophysin, calretinin, S-100, and HHV8. Ki-67 (MIB-1) showed an increased proliferation index of approximately 20% reflecting the number of EBV-positive atypical cells. Flow cytometry confirmed that the lymphoma cells were CD2 positive. There was cytoplasmic CD3 positivity, but there was no surface CD3 expression. Bone marrow aspiration and core biopsy revealed a normocellular marrow for age (30–40% cellularity) with active trilineage hematopoiesis and left shifted granulopoiesis but no evidence of lymphoma. Cytogenetic analysis did not reveal any clonal chromosomal abnormalities.

**Figure 3:**
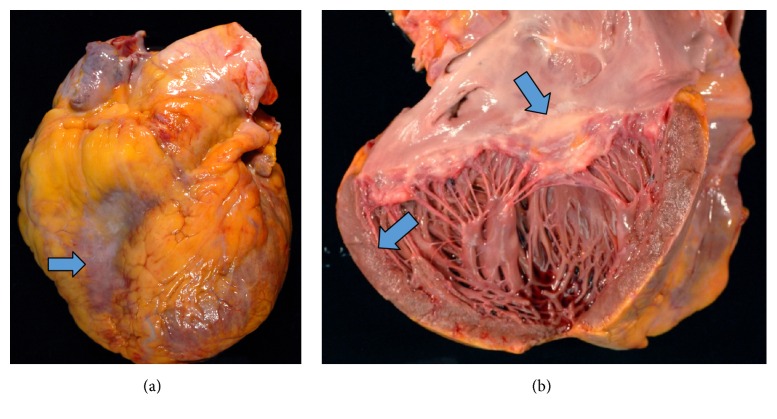
Macroscopic appearance of the heart at autopsy. (a) Cardiomegaly with whitish exudates on epicardium. (b) Dilated ventricles show patchy discoloration on endocardium and myocardium.

**Figure 4:**
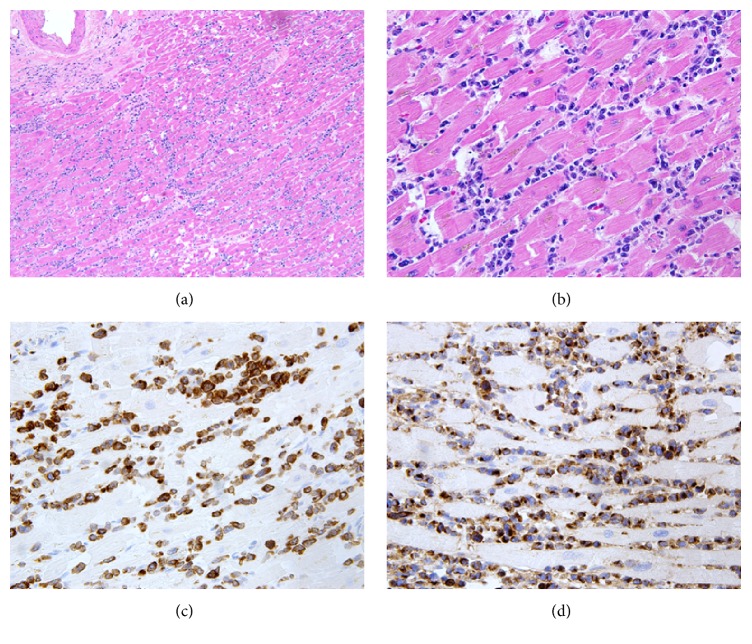
Microscopic appearance of lymphoma infiltrate in the heart. (a) Hematoxylin and eosin stain shows lymphoid cells infiltrating the myocardium (×100). (b) Medium sized atypical lymphoid cells with abundant pale cytoplasm, irregular nuclear contour, granular chromatin, and inconspicuous nucleoli (×400). (c) Lymphoma cells are immunohistochemically positive for CD3 with cytoplasmic staining pattern (×400). (d) Lymphoma cells are immunohistochemically positive for granzyme B (×400).

**Figure 5:**
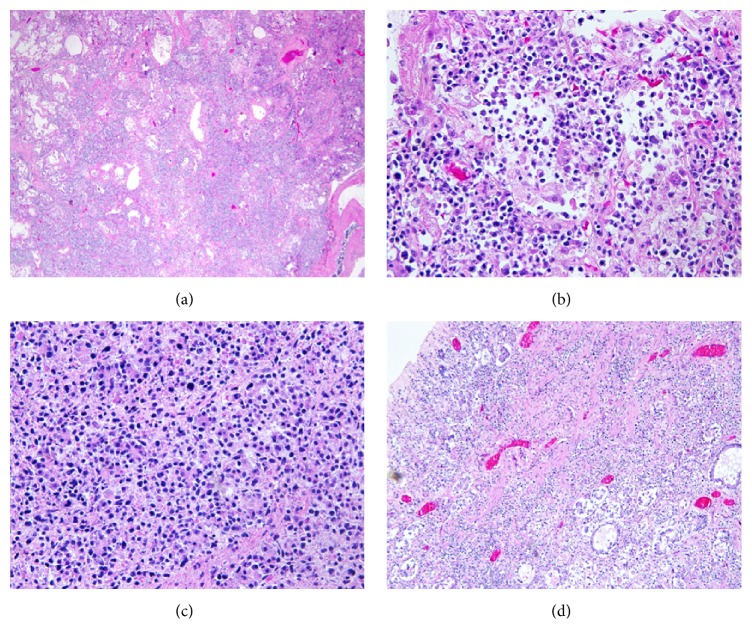
Lymphoma involvement of lung, pancreas, and stomach. (a) Diffuse alveolar and interstitial infiltrates of lymphoma cells in pulmonary parenchyma stained with hematoxylin and eosin (×100). (b) Higher power view of pulmonary infiltrates in a hematoxylin and eosin stained slide (×400). (c) Diffuse infiltrates of lymphoma cells in pancreatic tissue, showing architectural destruction in a hematoxylin and eosin stained slide (×400). (d) Transmural lymphoma cell infiltration in gastric tissue with mucosal erosion in a hematoxylin and eosin stained slide (×400).

At autopsy, the decedent was found to have cardiomegaly (weight 650 grams) (Figure 3(a)) with four-chamber dilatation and serous pericardial effusion (100 mL). Patchy whitish discoloration was found on the surface of epicardium and endocardium, and the myocardium had a mottled appearance on cross section (Figure 3(b)). The right and left lungs were heavy and weighed 1000 and 940 grams, respectively. The pleural surfaces were smooth, glistening, and unremarkable and the cut surface revealed patchy consolidation of pulmonary parenchyma with red and grey discoloration. There was 500 mL of straw colored pleural fluid in each of the pleural cavities. The pancreas was enlarged and the cut surface showed scattered areas of fat necrosis with hemorrhage. There was 500 mL of opaque fluid present in the abdominal cavity. The spleen was enlarged, weighed 330 grams, and had a smooth intact capsule covering red-purple semiautolytic parenchyma. The splenic white pulp was grossly unremarkable. Other organs were grossly unremarkable. Under microscopic examination, the myocardial tissue was found to be diffusely infiltrated by atypical lymphoid cells (Figure 4(a)). These cells had abundant pale cytoplasm, irregular nuclear contours, granular chromatin, and inconspicuous nucleoli (Figure 4(b)). In situ hybridization performed on the cardiac tissue showed that the atypical lymphoid cells were strongly positive for EBV. Immunohistochemical stains performed on the same tissue showed that the lymphoid cells were positive for CD3 (cytoplasmic stain) (Figure 4(c)) and granzyme B (Figure 4(d)) and negative for CD5, CD10, CD20, CD30, CD34, CD43, and CD56. Cells infiltrating the lung parenchyma in all five lobes (Figures 5(a) and 5(b)) had the same immunohistochemical features. Diffuse infiltrates of identical neoplastic cells were found in the pancreatic (Figure 5(c)) and gastric tissue (Figure 5(d)).

## 3. Discussion

ENKTL is a rare aggressive non-Hodgkin's lymphoma most prevalent in Asians and native populations of Central and South America. It is very rare in Europe and North America [1]. The ethnic background of our patient was thus very unusual for this form of lymphoma. Other unusual features were the lack of definitive evidence of sinonasal involvement and the predominance of cardiopulmonary symptoms. As shown in the head CT scan of our patient, there was only minimal mucosal thickening, opacification with fluid levels in nasal sinuses, without definitive neoplastic changes. At the time of admission, patient's chief complaint was cardiopulmonary failure and his nasal symptoms were inconspicuous. Based on the lack of mass lesion, erosion, or bone destruction, the imaging finding was considered to be inflammatory changes at least partially caused by the placement of nasoenteric tube. As mentioned by previous literatures, nasal infiltration by EBV-positive lymphoproliferative disorders or lymphoma can be meager and can show no significant gross abnormality. In a large cohort study by Gu et al., the imaging review demonstrated polypoid lesion only in 55.3% of cases of ENKTL nasal type [2]. The definitive diagnosis of lymphoma relies on biopsy; however the sensitivity and yield of nasal biopsies are apparently low in the absence of discrete radiologically visible lesions.

We are unaware of any other case of ENKLT presenting primarily with clinical signs of myocarditis and pneumonia as we have noticed in our case. Approximately 75% of ENKTLs occur in the upper aerodigestive tract, prominently involving the nasal cavity [1]. ENKTLs occurring outside the upper aerodigestive tract are often referred to as extranasal NK/T cell lymphomas. The most common sites of extranasal disease include gastrointestinal tract, skin, and testis [3]. The involvement of heart or lung with ENKTL is rare. Although there are reported cases of lung involvement by ENKTL [4–8], most of the lung involvements represent disseminated disease following nasal destruction. Only few cases are on record for presenting with primary lung involvement [7, 9]. Cardiac [10, 11] or pancreatic [12] involvement is extremely rare. Only one cardiac case and two pancreatic cases have been reported so far. Compared to sinonasal type, these extranasal ENKTLs are characterized by a rapid clinical downhill course, a poor response to treatment, and shorter survival time.

The tumor cells of ENKTL are usually classified as either T cells or natural killer (NK) cells; most often the tumor cells have a NK cell phenotype. The most typical immunophenotype is CD2+, CD56+, surface CD3−, and cytoplasmic CD3e+, with expression of cytotoxic molecules (such as granzyme B, TIA1, or perforin). Other T and NK cell associated antigens are usually not expressed [7]. Our case was negative for CD56 but had all other markers, which, according to the 2008 WHO classification of lymphomas, qualify it as an ENKTL. A molecular test to detect TCR gene rearrangement would be helpful for the determination of the cell of origin. However patient died shortly after the diagnosis was made and no additional samples were submitted for pathological analysis.

ENKTL is one of the lymphomas with clear association with hemophagocytic lymphohistiocytosis (HLH), which is a life-threatening severe hyperinflammation [13, 14]. In our case, patient had persistent fever, developed marked anemia, thrombocytopenia, elevated ferritin, and decreased fibrinogen when his condition started to deteriorate. The diagnosis of HLH was suspected clinically but the five criteria for the diagnosis of HLH could not be fulfilled because of patients' deteriorating condition, and the diagnosis remained tentative. Splenomegaly was identified at the autopsy, and the combined clinical and autopsy data meet the five criteria needed for the diagnosis of HLH. The cause of death was cardiopulmonary failure due to extensive involvement of heart and lungs by lymphoma.

ENKTL is a disease with strong association with EBV. Many studies have shown the significance of EBV DNA load in blood to predict the prognosis of ENKTL. In contrast, only one study was done to investigate the EBV load in tissue [15]. Interestingly our patient presented clinically with only 0.5 copies/*μ*L of EBV DNA which is low in comparison with other reported cases of ENKTL (average EBV load of 85.7 copies/*μ*L in whole blood) [16]. However EBV was diffusely and strongly positive in lymphoma cells infiltrating the organs at the autopsy. Patient's rapid disease progression implicated that the tissue EBV DNA load might be a more accurate indicator of his clinical stage and prognosis than the viral load in the blood.
